# “Let’s Talk about Physical Activity”: Understanding the Preferences of Under-Served Communities when Messaging Physical Activity Guidelines to the Public

**DOI:** 10.3390/ijerph17082782

**Published:** 2020-04-17

**Authors:** James Nobles, Clare Thomas, Zoe Banks Gross, Malcolm Hamilton, Zoe Trinder-Widdess, Christopher Speed, Andy Gibson, Rosie Davies, Michelle Farr, Russell Jago, Charlie Foster, Sabi Redwood

**Affiliations:** 1 The National Institute for Health Research Applied Research Collaboration West (NIHR ARC West), University Hospitals Bristol National Health Service Foundation Trust, Bristol BS1 3NU, UK; 2 Population Health Sciences, Bristol Medical School, University of Bristol, Bristol BS8 1TH, UK; 3 Knowle West Media Centre, Bristol, Bristol BS4 1NL, UK; 4 Mufti Games, Bristol BS5 6JL, UK; 5 Centre for Exercise, Nutrition and Health Sciences, School for Policy Studies, University of Bristol, Bristol BS8 1TH, UK; 6 Department of Health and Social Sciences, Faculty of Health and Applied Sciences, University of the West of England, Bristol BS16 1QY, UK

**Keywords:** physical activity, guidelines, communication, qualitative research, messaging, social marketing

## Abstract

Despite many countries having physical activity guidelines, there have been few concerted efforts to mobilize this information to the public. The aim of this study was to understand the preferences of under-served community groups about how the benefits of physical activity, and associated guidelines, can be better communicated to the public. Participatory workshops, co-developed between researchers, a local charity, and a community artist, were used to gather data from four groups in Bristol, UK: young people (*n* = 17); adults (*n* = 11); older adults (*n* = 5); and Somali women (*n* = 15). Workshop content was structured around the study aims. The community artist and/or the local charity delivered the workshops, with researchers gathering data via observation, photos, and audio-recordings, which were analysed using the framework method. All four groups noted that the benefits of physical activity should be included within any communications efforts, though not restricted to health-related benefits. Language used should be simple and jargon-free; terms such as “sedentary”, “vigorous” and “intensity” were deemed inaccessible, however all groups liked the message “some is good, more is better”. Views about preferred mechanisms, and messenger, for delivering physical activity messages varied both between, and within, groups. Recommendations for those working in physical activity communications, research, and policy are provided.

## 1. Introduction

In 2019, the four Chief Medical Officers (CMO) of the United Kingdom (UK) published updated guidelines for physical activity. These were first introduced in 1996 and updated in 2004 and 2011 [1]. The guidelines, which are based on epidemiological thresholds, offer recommendations for the volume, duration, frequency and type of physical activity that individuals should do to significantly reduce the risk of a range of conditions, diseases and mortality [1]. Specific guidelines were updated across population age groups, including children and young people (under 5 years and between 5–18 years), adults (19–64 years), and older adults (65 years+). Further guidelines have also been developed for adults with a disability, and for women during and after pregnancy [1]. To support the dissemination of such guidelines, the CMOs published updated supplementary infographics, which aim to summarize key components of each guideline for healthcare and relevant professionals [2].

Physical activity is associated with improved physical health, mental health, workplace productivity, and in turn, a more prosperous economy [3]. In short, physical activity is beneficial, with positive effects immediately obtainable and from small bouts of activity [1,4,5]. Yet, epidemiological studies in England, such as Sport England’s Active Lives survey (an annual survey distributed to a representative sample of approximately 200,000 adults) suggest that almost four in 10 adults do insufficient physical activity, with 25% of the population deemed to be “inactive” (less than 30 min per week) [6]. These findings are mirrored in the Health Survey for England [7]. The Active Lives data also illustrate issues of inequality; the prevalence of inactivity in areas of greatest deprivation is double that of the least deprived areas (33% and 16% respectively) [6].

Population level physical activity is influenced by many multifaceted and complex factors [3,8]. Low levels of physical activity are the product of a complex adaptive system, whereby the environments in which people live are designed in way that promotes a sedentary lifestyle–from workplaces, to transport options, education settings for young people, to the social groups with which we interact. Thus, in order to increase population levels of physical activity, members of the public cannot be expected to be the sole agents of change. A comprehensive, multi-sector, systematic and aligned approach is required to alter the systems in which we live [3,8]. Physical activity guidelines, and the communication of guidelines to the public, provide only part of this solution [9,10]. There is some evidence to suggest that social marketing and mass media campaigns which are focused on physical activity messaging can affect awareness, attitudes and intentions, and physical activity behavior, when implemented within a comprehensive approach [9,11,12,13,14,15] albeit that individual intervention effects are difficult to isolate within such multi-component approaches [15].

Despite many countries having evidence-based physical activity guidelines for up to the last 20 years, public knowledge and awareness of key parts of guidelines is very limited. For example, Knox et al. [16] surveyed 1724 adults and found that 18% were able to accurately recall the UK 2011 CMO guidelines for moderate-to-vigorous physical activity (i.e., the aerobic guideline—150 min per week). Similarly, Hunter et al. [17] in a cross-sectional and representative survey of 4653 Northern Irish adults, reported a smaller proportion of the sample (8.4%) correctly identified the moderate-to-vigorous physical activity recommendation from a list of 17 options. Those less likely to know the guidelines tended to be males who had lower levels of education, low income, lived in more deprived areas, and did no physical activity–starkly illustrating health inequalities. There are similar findings from other countries [18,19,20,21,22]. Nationwide campaigns have sought to increase public awareness of physical activity, its benefits, and the associated guidelines–e.g., Change4Life (UK [23]), Find Your 30 (Australia [24]), ParticipACTION (Canada [25]), and VERB (America [26]). However, physical activity guidelines–in and of themselves–are not necessarily intended for public consumption [27], they are written for professionals, practitioners, and policy makers [1]. Thus, greater efforts are required to mobilize this knowledge to the public.

Messaging is a key component of communicating guidelines [28]. In the context of this paper, messaging is a form of communication, whereby information is conveyed to an audience in order to promote changes in physical activity patterns for health-related benefits [28]. Brawley and Latimer [29] go on to suggest that physical activity guidelines should be *translated* into physical activity messages, and that these messages are informative, provocative, persuasive and influence the determinants of physical activity (e.g., self-efficacy, motivation, etc.). In a recent review of the evidence-base surrounding physical activity communications in the USA (*n* = 67 included studies), Bergeron et al. [30] found that communications were predominantly aimed to persuade the public to be physically active (95.5% studies) or to increase their self-efficacy in physical activity (61.2%). In addition, one third of the communications efforts included the national physical activity guidelines, with the message content primarily emphasizing the health benefits associated with physical activity (89.6% studies). These physical activity communications were mostly conducted through mass media campaigns (65.7% studies). The review also identified substantial knowledge gaps, one of which is the volume of qualitative research that explores the social, cultural and contextual factors related to physical activity communication, particularly in under-served population groups, and who–as mentioned beforehand–are less likely to be physically active [30].

The aim of this study was to understand the preferences of under-served community groups (i.e., those who live in areas which are more deprived of resources: e.g. stable, well-paid jobs, quality housing, access to healthcare, etc.) for how physical activity messages, and associated guidelines, can be better communicated to the public. We focused on two questions: (1) what do under-served community groups in Bristol (West of England, UK) understand physical activity to be; and (2) how could physical activity guidelines, and physical activity messages more broadly, be communicated to these community groups? Based on these findings, we sought to develop a set of practical recommendations that can be used by health professionals, policy makers, and researchers to improve how physical activity information is mobilized effectively to under-served groups.

## 2. Materials and Methods

The study employed a qualitative participatory design to explore the views of people living in under-served communities in Bristol, UK. The study team was a collaboration of researchers from the University of Bristol, staff from an arts-led community-based charity (Knowle West Media Centre, KWMC) and a community artist (Mufti Games). The research project was also underpinned by the Bristol Approach; a six-step framework developed by KWMC to describe a way of working to support communities to come together to tackle pressing issues affecting their lives [31]. The steps of the cyclical framework involve; *identification* of the key issue for change, *framing* the issue in more detail, *designing tools* to address the issue, *deploying the tools* in the real world, *orchestration* in order to share tools and *celebrate achievements* and evaluation of outcomes, which then feeds back into a new cycle starting with identifying issues for change. The study received research ethics committee approval from the Faculty of Health Sciences, University of Bristol (Ref. 80322).

### 2.1. Participatory Workshops

We delivered four participatory workshops during March 2019 (workshop lasted two hours), each with a different group of people who live in under-served areas of Bristol. Groups were split into the following: children and young people (aged under 18 years), adults (aged 19–65 years), older adults (aged over 65 years), and Somali women (aged 19–65 years). Novel workshop activities were designed by Mufti Games in collaboration with KWMC and the researchers. The workshops were facilitated by Mufti Games and/or KWMC engagement workers and used various playful activities and props (such as bells and balloons) to stimulate discussion. Several activities included an element of movement to align with the theme of the workshop. The workshop was designed to be informal, interactive and enjoyable, and reflected a conscious effort of the study team to move away from traditional qualitative methods (e.g., interviews and focus groups) with the aim of engaging people who may normally not respond to invitations to participate in traditional methods.

The workshop was split into four interlinked sections: (1) understanding group perceptions of physical activity; (2) knowledge and awareness of the UK physical activity guidelines and other physical activity campaigns (ThisGirl Can and Change 4 Life); (3) group feedback on the UK physical activity guidelines relevant to their age group; and (4) developing a physical activity message suited to group preferences. The fourth section was informed by the physical activity messaging framework of Williamson et al., [28]; as such this section sought to establish group preferences regarding message content (i.e., what should go into the message), language (i.e., the terminology and tone of the message), the messenger of the information (i.e., who should be used to promote or disseminate the message) and the mechanisms for delivering the message (i.e., how to disseminate the message to the public). The first and second sections were conducted as a whole group; for the third and fourth sections, participants worked at tables in smaller groups. An overview of the workshop plan, including methods and approximate timings, is available in online Supplement I. The core components of the workshop were delivered consistently across the four groups, but several minor modifications were made to suit the preferences of the four groups (e.g., seated activities with older adults and using female facilitators with the Somali women’s group). Workshops were delivered local community venues that were familiar to participants.

### 2.2. Participants

Workshop participants were recruited by a KWMC engagement worker through established contacts with individuals and community groups within local under-served communities. Participants were provided with written information about the research, had an opportunity to ask questions and written informed consent was obtained from all. Participants were given a £20 shopping voucher for taking part in the workshop. Eleven adults, five older adults, 17 young people and 15 Somali women took part in the workshops. Table 1 provides information about the demographic characteristics of the participants at each workshop.

### 2.3. Data Collection and Analysis

Two researchers attended each workshop to gather data. The researchers observed the workshops, independently taking field notes which covered the discussion content and contextual factors such as group dynamics and facilitation, using a data collection form (online Supplement II). In some instances, researchers asked probing questions to elicit further information. The workshops were audio recorded (in Section 3 and Section 4 of the workshop, a dictaphone was placed on each table), photographs were taken of activity outputs and the facilitators collated and typed up what had been written on post-it notes by participants during the workshop. After the workshop, a researcher merged the facilitator’s notes and the two sets of fieldnotes, then listened back to the audio recordings to add any further information, thus creating a single comprehensive account of participants’ views and researchers’ reflections for each workshop. These four accounts made up the data set for analysis. Analysis of the data followed the framework approach described by Gale et al. [32]. We took a broadly deductive approach to the development of the analytic framework by structuring it around the four key domains of physical activity messaging [28]. We were particularly interested in comparing and contrasting the views expressed by the four groups within these key domains, which was facilitated by the process of charting the data into a framework matrix. Two researchers (J.N. and C.T.) analysed the data using the framework matrix, with any uncertainties or disagreements being discussed until consensus reached.

## 3. Results

The findings of the study are divided into five key themes. The first theme relates to perceptions and value of physical activity including what participants understood physical activity to be and its benefits. The remaining four themes relate to their preferences in different domains of physical activity messaging, namely: (1) message content; (2) language used within the message, (3) the messenger of the physical activity message, and (4) the mechanism of delivering the physical activity message. Within the text below, we identify the key similarities and differences between the four groups. An overview of the results, and excerpts from the observational field notes, is provided in Table 2.

### 3.1. Perceptions of Physical Activity

When asked what the phrase “physical activity” means to them, participants gave examples of both intentional physical activity, such as sports, exercise, play or recreational activities, and incidental physical activity, such as active travel, housework, gardening and shopping. For the most part, groups were more likely to mention intentional physical activities initially, and the extent to which incidental activities came to mind without prompting or were seen to “*count*” as physical activity varied between individuals and across groups. For example, the following was noted by a researcher in the Somali women’s group “*Many don’t see the activities they already do (*e.g., *school run, housework, shopping) as ‘physical activity’*”, and instead they often perceived physical activity to be high intensity intentional activities. The participants talked about the benefits of physical activity in terms of physical and mental health and well-being, social connection, appearance/weight loss, energy and enjoyment. Physical activity for health benefit or in response to a health scare were more pertinent to older participants, but the impact of mobility issues and other health problems on ability to participate in physical activity was also highlighted. The young people and younger adults generally placed more emphasis on the enjoyment, well-being and mental health benefits, with physical activity being described as “*providing a feel good factor*”. Physical activity as an opportunity for social connection was seen as important by all groups, but conversely for some of the mothers in the Somali Women’s group physical activity was also seen “*as a means of having time to yourself*”. Benefits relating to appearance or weight loss were mentioned but were generally seen as less important.

As this extract from the older adult’s workshop demonstrates, ideas about the most important benefits sometimes evolved through the course of the discussion.

*“In the first activity…the group believed the health benefits to be the most important. However, as we moved towards the latter activities…the socialisation aspects prevailed as the most important…the group all agreed that in hindsight, they did think that the socialisation/reducing loneliness was the most important benefit of physical activity, and that the health improvements were secondary.”*

### 3.2. Preferences for Physical Activity Messaging Content

Ideas about message content were largely consistent across the four groups. Physical activity messages should include information about the benefits of physical activity, with emphasis given to mental health, enjoyment and opportunity for social connection as well as physical health benefits. Messaging should make recommendations feel achievable and realistic, focusing on progression towards a guideline rather than on the achievement of the guideline per se. These views are exemplified by these extracts from the adults’ workshop: “*Why couldn’t they make it about having fun though, rather than saying you have to do 150 min a week?*” and “*Like the phrase ‘any activity is better than none’—should come first in the guidelines*”.

All groups reported that providing examples of physical activity at the different intensities would be helpful, in particular highlighting how physical activity can be incorporated into daily life or take account of pain, mobility or other health issues. Physical activity messages should also challenge the assumption that being physically active requires playing sport or going to the gym, for example: “*…point out the things you do as part of day to day life that constitute physical activity… [that don’t] cost money and time–things which people don’t have*” (Adults’ workshop).

Participants also suggested that where images of people are used, it is imperative that they are relatable (i.e., they look, talk, and have similar experiences to the message recipient): “*normal people-selection of different people, of all different ages and backgrounds, doing different types of activity as part of normal life—this should be the imagery in the campaign*”. Participants also suggested that the content be “*motivating*”, “*encouraging*” and “*light-hearted*”.

For the Somali women, the content also needed to demonstrate an understanding of cultural sensitivities, as this extract shows: “*Because of my religion, I would not be able to exercise in the park, or wear revealing clothes, it has to be inside. Indoor exercise, with only ladies and a female instructor would be the only way. [Also] having an instructor who understands that religious clothing would still need to be worn when exercising*.”

### 3.3. Preferences for Physical Activity Messaging Language

All groups reported that the language used should be simple, non-academic and jargon-free, highlighting the need for the ‘translation’ of the language used in the UK guidelines, which was largely percieved as inaccessible by all four groups. Words such as aerobic, intensity, and sedentary were identified as words to avoid, as demonstrated through these quotes and observational excerpts:

*“I thought sedentary was some kind of rock.”*

(Children and young people’s workshop)

*“Didn’t understand the word aerobic–associated with jumping around in a leotard.”*

(Older adults’ workshop)

*“No idea what ‘intensities’ are.”*

(Adults’ workshop)

*“Words like moderate, vigorous, intensity, aerobic are scary/frightening.”*

(Older adults’ workshop)

Three of the groups (children and young people, adults and older adults) suggested that messages should be invitational rather than instructive (terminology used by the community artist), avoiding the sense of being told what to do. The children and young people wanted language that was “*snappy*”, “*chatty*”, “*friendly*” and “*encouraging*”, and along with the older adults, recommended using humour. By contrast, some of the Somali women said that more instructive, direct messaging would be necessary to influence motivation to be more physically active. When critiquing the UK guidelines, all groups stated that the message, “*some is good, more is better*”, struck the right tone.

### 3.4. Preferences around the Messenger

There was variation between groups, and in some cases within groups, in preferences around the messenger. Whilst all groups highlighted the need for the messenger to be trustworthy and influential, the type of people they believe had these characteristics differed. For example, the Somali women perceived that healthcare and physical activity professionals were key influencers, whereas the children and young people mentioned celebrities, sports stars and YouTubers (many of whom the young people could relate to).

*“Celebrities were seen as the best messenger of this information. Group believes what celebrities say to them/show them. For example, if they see a celebrity being active or talking about activity, they assume that the celebrity is active themselves.”*

(Children and young people’s workshop)

There were mixed views about celebrities in the adults’ and older adults’ groups, with some finding celebrity endorsement persuasive, whilst others questioned how relatable and trustworthy they are.

*“If it comes from someone who is really attractive, really fit, you think ‘they don’t work a real job’, you just work out all day, I have got to go to work…I don’t have an army of people doing all my chores.”*

(Adults’ workshop)

*“Celebrities are perceived favourably. Noted that it is good to see Serena Williams in the Nike advert, and to see footballers doing work in the community around knife crime…”*

(Older adults’ workshop)

That messengers should be relatable was again a key finding; messengers should represent “*everyday people, people like us*.” (Adults’ workshop) and include people of different ages, genders and cultures. Friends and peers were mentioned, in particular, by younger participants and by the older adults and community and social networks by older adults and Somali women.

### 3.5. Preferences Around Mechanisms for Delivery

The groups suggested that a variety of mechanisms are needed to deliver the message. All groups preferred visual mechanisms, and suggested that multiple mechanisms (e.g., social media, TV, posters) be used to both increase the reach of the message (due to seeing the message in many places) and to reinforce the message (due to a consistent message being communicated). Social media channels (such as Facebook, Twitter, Instagram and Snapchat) were mentioned by all except the older adults. The younger participants also highlighted YouTube, streaming platforms, podcasts, apps and video games as potential vehicles for the message. Adults and older adults mentioned eye-catching posters, on billboards and bus stops and leaflets available in libraries or distributed though schools or in healthcare settings. Utilising local advertising opportunities, such as community noticeboards or newsletters was highlighted by the Somali women in particular. Radio and TV were also mentioned, but with mixed views about their reach. The use of inspiring and emotive personal stories was a suggested mechanism for communication by adults.

*“Need to have a campaign that can be seen everywhere–in the places that people are (TV ad, posters/billboards, social media). Posters on the bus stops—‘everyone sees bus stops’. Not everyone goes to the doctors, not everyone has a TV. Need to cater for multiple audiences.”*

(Adults’ workshop)

*“Some thought TV would be a good place to put the message (e.g., having someone being interviewed on the sofa on Good Morning TV) but others said they didn’t take much notice of TV.”*

(Older adults’ workshop)

## 4. Discussion

This study explored how members of the public, from under-served areas in Bristol, would prefer physical activity messages, and the new 2019 UK physical activity guidelines, to be communicated to them. The results demonstrate commonalities and differences between the four groups regarding their messaging preferences. We have used our findings to propose several key recommendations for those wishing to communicate physical activity messages to the public, and particularly to groups who are most likely to be amongst the least active in the UK.

### 4.1. What Should Be Included in A Physical Activity Message?

Getting the content of a physical activity message right is imperative. Our findings indicate that a physical activity message should include the benefits of being physically active, but, as other research suggests, these benefits must go beyond those relating to physical and mental health improvements, to also include social benefits. Across most groups, enjoyment and the opportunity to socialize, were highly regarded benefits of physical activity, and all groups suggested that these benefits are key content for a physical activity message, which Bergeron et al. [30] and Latimer-Cheung et al. [33] refer to as affective benefits. 

These findings are consistent with framing theory [34], and highlight the need to use gain framing within message content. This type of framing emphasizes the benefits that are to be gained when carrying out the promoted behavior (e.g., improved health, social connectedness etc.) as opposed to focusing on the harms that may arise if the behavior is not undertaken (i.e., loss framing). Gain framing has been evidenced within the physical activity literature to bring about favorable outcomes [27], although the broader evidence base around framing effects is inconclusive [35], thus care should be taken not to overinterpret this finding, and its subsequent effectiveness for physical activity messaging.Social marketing provides an evidence base in which to situate some of our findings, both in terms of physical activity message content, and more broadly, to message development. Alan Andreasen [36] created what are known as the social marketing “benchmark criteria” (Table 3), which have been widely applied to evaluate and synthesize physical activity promotion efforts [13,14,37]. Our findings point towards the importance of four of these criteria. First, message content preferences differed between our four groups, and as such highlights the need for future studies to conduct formative research before developing physical activity messages. This also links to the second point, that formative research should help identify and characterize sub-groups within a population based upon their “*needs*” and “*wants*”. Such segmentation will enable message content to be tailored to the preferences of various population segments. Third, by outlining their perceived benefits of physical activity, participants provided information that can help create an attractive and motivational physical activity message, so much so that it prompts behavior change (i.e., an exchange). Whilst the UK physical activity guidelines and respective infographics use gain framing by including the health benefits associated with increased physical activity, those creating messages in the future should also emphasize the wider benefits of physical activity (e.g., social connections) which are salient to specific audiences. And lastly, two groups (Adults and Somali women) said that the message should demonstrate how physical activity can fit into people’s daily lives, rather than having to compete for time against other daily priorities. This may increase individual motivation to undertake physical activity [38]. Many of the recommendations generated by the social marketing literature are also echoed within the broader health communications literature, for example, see Noar [39] and Wakefield et al. [15].

Another key consideration regarding message content is whether to include the physical activity guidelines in the message (e.g., 150 min of moderate-to-vigorous physical activity per week). Our findings are consistent with other studies which suggest that the guidelines can appear unobtainable, and therefore their inclusion may be demotivating [33,40,41,42,43]. Our findings also suggest that reference to different intensities of physical activity (e.g., light, moderate, vigorous) is problematic, as these terms were not well understood by the four community groups. If intensities are to be included in a physical activity message, participants asked for examples of various activities to be provided to illustrate *what* ‘moderate-to-vigorous’ looks like and *how* it can be achieved—a finding echoed by others [43]. If the aim of a physical activity message is to encourage behavior change, then ensuring that message content is understandable, salient and persuasive is perhaps more important than inclusion of the recommended physical activity dose [16,20,27,29]. This is a consideration that physical activity communicators should be mindful of.

### 4.2. What Language should Be Used in A Physical Activity Message?

We sought to understand what language and tone was appealing to the four community groups within physical activity messages. All groups stated that they wanted simple, jargon-free language to be used, and a tone that does not challenge their sense of autonomy. Whilst the UK guidelines are not intended for public consumption, they often end up being communicated to the public (e.g., Health Education England [44] p.8, and being cognizant of what is, and is not, appealing within these guidelines is important. Terms such as “*sedentary*”, “*intensity*”, and “*muscle-strengthening*” were not understood by many, and the tone was perceived by all as instructive and/or clinical. Three of the groups believed this tone to be ineffective due to the attached connotations that the message originates from a Government organization. On the other hand, the statement “*some is good, more is better*” was viewed as simple and understandable, and similar to examples of Latimer-Cheung et al. [33], it focuses on general movement rather than physical activity of a specific type or intensity. Furthermore, care should be taken when creating messages to ensure that physical activity is not conflated with exercise and sport–as Faulkner et al. [43] also notes. This conflation is problematic given that exercise and sport are intentional, structured activities; if this is a dominant public perception for what physical activity is, then it is not surprising that the recommended 150 min per week seem unobtainable. Thus, the language used requires careful thought; Faulkner et al. [43], via consultation with 104 stakeholders, reported that the main barrier preventing stakeholders from understanding the guidelines was the language used.

### 4.3. Who Should Communicate the Physical Activity Message to the Public and How Should This Be Done?

Two other key concepts were explored within this study; the messenger and the mechanism for delivering the physical activity message. Whilst there was a variety of opinions within, and between, groups as to who the messenger(s) could be, it was important to all that they were deemed as trustworthy, influential and relatable. Berry et al. [45], Latimer-Cheung et al. [33] and Faulkner et al. [43] concur. For children and young people, and for some older adults, celebrities were seen as effective messengers because of their perceived status and influence in society. However, as Berry et al. [45] notes, these celebrities may still need to “look like me” in order for the message to resonate with the public. But for many adults, older adults and the Somali women, social and community groups were key messengers too-exemplifying the importance of relatedness and the mechanism of word-of-mouth. Thus, identifying and engaging influential and trusted individuals and organizations within local communities may be a key mechanism for delivering a message. It is clear from working with these four groups that formative research, as advocated by Andreasen [36] and others [39,43,46], should be a key requirement for any physical activity messaging work.

Lastly, our results indicate that the groups had inconsistent preferences regarding the mechanism of delivery. It is likely that some mechanisms may work better than others, linked into the influence and trustworthiness of the messenger, but it also reinforces the need for a comprehensive, multi-modal approach to physical activity messaging (i.e., social media, TV, radio, billboards, flyers, word-of-mouth). Across the literature, this is a recurrent finding [27,39,43,45,47], so it is therefore imperative to create an attractive message–which can be promoted via multiple mechanisms–in order to increase individual motivation to undertake a behavior [38,47]. However, in light of the proportionally smaller budgets in the public sector, compared to the commercial sector, Berry et al. [47] suggests that public sector and voluntary sector organizations pool resources to maximize the likely impact of the physical activity message or campaign. A collective message may also help to reduce public confusion around physical activity due to conflicting and contradictory information.

### 4.4. Recommendations for Physical Activity Communication

This study, as part of its aims, set out to create a series of practical recommendations for those who communicate physical activity messages, and the guidelines, to the public. Due to the groups that we worked with, our findings may also be particularly relevant when aiming to develop messages for people who live in under-served communities. Table 4 provides a summary of our main recommendations, which are also offered as an infographic in online Supplement III.

## 5. Strengths and Limitations

This study was designed to capture the views of under-served community groups in Bristol, UK. In doing so, colleagues from ARC West worked in collaboration with KWMC (an arts-led community-based charity) to effectively engage a diverse range of community members. Given that three of the four workshops were well attended, particularly those with younger people and Somali women, suggests that we successfully engaged these groups. This success underlines the importance of carefully co-designing these types of physical activity with community-based organizations. It is unlikely that the sample of participants would have been recruited as efficiently without the expertise of KWMC. Similarly, in working collectively with KWMC, we were able to commission a local art organization, Mufti Games, to help design and deliver the participatory workshops. This three-way collaboration enabled the team to develop creative and novel methods to gather data from workshop participants—a major strength of the study.

The study also has two main limitations that should be recognized. First, we had fewer older adults attend their respective workshop than anticipated (*n* = 5)-the reasoning for which is unclear. To understand whether the findings from this workshop were reflective of other older adults in Bristol, we ran a public involvement event (a 2-h conversation guided by the main research questions) with a demographically similar group (*n* = 9; 6 female). Broadly, the information ascertained from the involvement event was in line with that of the study participants, however they suggested that TV was not an important mechanism for physical activity messaging, that celebrities were therefore not an important messenger, and lastly, that improving mobility was also an important benefit to consider. We hope that this public involvement increases the trustworthiness of our findings with regards to the preferences of older adults. Second, we also note that the four groups are not homogenous; a sentiment which members of the older adult’s group also expressed, and we did report several within-group differences in our findings. This limitation reinforces the need for future work to consider the use of segmentation, and to inform this segmentation through high-quality formative evaluation.

## 6. Conclusions

Physical activity messaging is an important component of a comprehensive approach to increasing population physical activity levels. However, given that poor physical activity messaging may cause disengagement amongst the public, the need to ensure public resources are well spent on effective communications efforts is pressing. Through working with four community groups, and in hearing the voices of people who may not engage in traditional research methods, our study illustrated how the updated CMO physical activity guidelines can be mobilized to design messages that are aligned with the motivations, experiences and goals of different population groups. We also demonstrated the value of including target recipients in scoping out physical activity messaging preferences; it is suggested that target recipient involvement will improve the outcomes associated with such campaigns. Our findings emphasized the importance of tailoring message content and language to the needs of different audiences, using a wide array of messengers and delivery mechanisms. Although many of our conclusions align with the existing literature base, this study has provided several new insights around physical activity messaging to under-served community groups. We hope that the findings of this study will inform future work around the communications of physical activity and physical activity guidelines. 

## Figures and Tables

**Table 1:** Demographic characteristics of workshop participants.

Demographic Characteristic		Adults (*n* = 11)	Older Adults (*n* = 5)	Young People (*n* = 17)	Somali Women (*n* = 15)
Gender	Male	9.1	0	47.1	0
(%)	Female	81.8	80.0	52.9	100.0
	Prefer not to say	9.1	20.0	0	0
Age	Mean	38.2	73.2	12.2	32.0
(years)	Range	23–57	67–87	10–15	18–55
	Prefer not to say (*n*)	1	0	0	4
Ethnicity	White British	54.5	100.0	88.2	0
(%)	Other Ethnicity	36.4	0	11.8	73.3
	Prefer not to say	9.1	0	0	26.7

**Table 2:** Summary of key results.

	Children and Young People (*n* = 17)	Adults (*n* = 11)	Older Adults (*n* = 5)	Somali Women (*n* = 15)
**Perceptions of Physical Activity**				
**Interpretation and benefits of physical activity**	**Physical activity perceived as**: *Intentional physical activity*: (1) sport, and (2) play and recreation. *Incidental physical activity*: 1. travel, and 2. chores. **Benefits of physical activity included ^1^**: (1) Enjoyment and wellbeing, (2) appearance, and (3) social connections.	**Physical activity perceived as**: *Intentional physical activity*: (1) recreational, and (2) sport. *Incidental physical activity* (occupational, chores, gardening). **Benefits of physical activity included ^1^**: (1) Mental health, (2) fitness, (3) weight loss, (4) social connections, (5) energy, and (6) competition. Younger participants likely to do physical activity for enjoyment, older participants for health benefit. Long-term health condition has negative impact on physical activity participation.	**Physical activity perceived as**: *Intentional physical activity*: (1) sport, and (2) recreational. *Incidental physical activity* (chores, gardening, shopping). **Benefits of physical activity included ^1^**: (1) Physical health, (2) wellbeing, (3) social connections, and (4) enjoyment. Social connections became more pertinent throughout conversation. Perceived as more important after a “health scare”.	**Physical activity perceived as**: *Intentional physical activity*: (1) sport, and (2) recreational. Walking seen as physical activity but to a lesser extent. Physical activity should be high intensity. Physical activity for younger generation, seen as aspirational by older generation. **Benefits of physical activity included ^1^**: (1) Physical health, (2) mental health, (3) appearance, (4) energy, (5) social connections, and (6) enjoyment. Perceived as something fun to do with children.
**Communicating Physical Activity Messages to the Public-Preferences**				
**Content**	Include: (1) The benefits of physical activity. (2) Examples of physical activity and at differing intensities. (3) Physical activity ≠ sport. (4) Humorous and/or light-hearted content. (5) Images of people having fun. (6) Statements that focus on “building up to the recommendations”.	Include: (1) Focus on positive emotions linked to physical activity (e.g., enjoyment). (2) A call to action–inspire and motivate people to be more physically active. (3) Inclusive messages that appeal to multiple audiences (i.e., those with lower levels of physical activity). (4) Statements on “building up to the recommendations”. Do not focus on the guideline per se. Needs to feel obtainable. (5) Examples of what can be done in daily life (i.e., incidental physical activity). Make people aware that they likely already do incidental physical activity. Avoid examples that sound overly strenuous. (6) The benefits of physical activity (demographic specific). (7) Images of “people like me”.	Include: (1) The benefits of physical activity. Social connections, health and enjoyment. Emotive content. (2) Realistic recommendations. Build up levels of physical activity. Don’t need to focus on the guideline per se. (3) Examples of different types of physical activity. Must account for physical limitations (e.g., chair-based activity). (4) Relatable imagery. Make people believe that physical activity is realistic for them.	Include: (1) Statements that focus on “building up to the recommendations”. Guidelines represent a target (negatively perceived). (2) Encouraging content. (3) The benefits of physical activity (“feeling good” and “time with family”). (4) Examples of what constitutes physical activity (including incidental physical activity). (5) Content that is mindful of cultural differences. (6) Relatable images and use of role models.
**Language**	(1) Simple and understandable. (2) Invitational—“don’t want to be told what to do”. (3) “Chatty, friendly and encouraging” tone. “Snappy, catchy” language. (4) Avoid CMO guidelines language—academic, clinical, inaccessible. “I thought ‘sedentary’ was some kind of rock”—CMO guidelines feedback.	(1) “Some is better than none” and “Just do a little bit of anything” (2) Invitational rather than instructive (e.g., we did it, so can you). (3) Be aware of negative connotations (e.g., muscle strengthening was associated with body building). (4) Avoid CMO guidelines language—not suitable for the public. Remove jargon. Simple. (5) Similar to the C4L language.	(1) Invitational language (e.g., how are you doing? Fancy a brew?). Link with emotive content. (2) Light-hearted and humorous. Needs to be encouraging. (3) CMO guidelines— “some is better than none”. (4) Avoid CMO guidelines language—not suitable for the public. Remove jargon. Simple. “Don’t want to google the words”—Feedback on CMO guidelines.	(1) Instructive messaging may be needed for some. (2) CMO guidelines— “Some is better than none”. Needs to be encouraging and realistic. (3) Remove all jargon. “This is BBC language, not ours”—Feedback on CMO guidelines.
**Messenger**	Messenger must be perceived as important and trustworthy. Messenger should also embody the promoted behavior. Messengers included: - Celebrities (differ depending on age group) - Sport stars - YouTubers and gamers - Friends and peers	Messenger must be perceived as influential. Demographic specific. Relatable-“People like me, everyday people in everyday settings”. Younger adults influenced by friends and peers. Older adults influenced by authoritative figures (e.g., doctors). Messenger could look like they have benefitted from physical activity. Not celebrities—unrelatable.	Messenger must be perceived as trustworthy and caring—“A kind voice”. Someone with real-life experience. Friendship groups and community important messengers—social networks. Celebrities well perceived amongst many. Specific to demographic. Mixed perceptions of public sector professionals (trustworthiness). Need to speak in lay language.	Messenger must be relatable. Different people of different ages. Healthcare and physical activity professionals perceived as influential.
**Mechanism**	Differs dependent on participant’s age: - *Younger*: Visual (TV, YouTube), catchy music and lyrics, snappy text. - *Middle*: TV, podcasts, attractive imagery. - *Older*: Streaming platforms, social media, radio, video games, apps, and GIFs. Note: whilst many raised TV as a mechanism—many stated that they don’t watch terrestrial TV. Social media noted by all.	Different mechanisms for different audiences. - Posters—colorful, clear/simple text. Child friendly–light-hearted. - Stories seen as powerful. - Imagery important (people being active—show progress and benefits). - Other: TV, radio, billboards, bus stops, apps, and social media. Needs to be visible in many places. Avoid government websites.	Mechanism must be simple, primarily visual. Easy to understand. No agreement on mechanisms between group. TV perceived as useful by some, not all.	Social media seen as main mechanism–different platforms (Twitter, Facebook, Instagram, YouTube, snapchat). Including community forums. Social groups and social media useful mechanisms to disseminate. Local advertising (e.g., community groups, leaflets and newsletters, bus stops, healthcare settings). Online articles, TV and radio also mentioned.

**^1^** The ordering refers to the perceived importance of the benefits of physical activity. BBC—British Broadcasting Corporation, C4L—Change4Life, CMO—Chief Medical Officer, GIF—Graphics Interchange Format, TV—Television.

**Table 3:** Andreasen’s Social Marketing Benchmark Criteria.

Benchmark Criteria	Definition ^1^
1. Behavioral objective	Social marketing should aim to change or focus on a specific behavior of the target recipients (e.g., physical activity).
2. Formative research	Formative research should be undertaken to learn about the target audience in order to shape the intervention/message being developed (e.g., understanding the determinants which influence physical activity behavior amongst the target recipients).
3. Segmentation	Segmentation acknowledges that similar groups lie within heterogenous populations, and can be identified based upon their “needs” and “wants” (not necessarily based upon their demographic make-up). Information can be tailored to these segments.
4. Exchange	It is important to consider what would motivate target recipients to voluntarily take up the desired behavior–may be intrinsic (e.g., enhanced sense of wellbeing) or extrinsic (e.g., financial incentive) motives.
5. Marketing mix	Social marketing strategies–including communication and messaging–should consider the four Ps of marketing: product (i.e., the benefits received if undertaking the behavior), price (i.e., the opportunity costs when carrying out the behavior–pros and cons), placement (i.e., where the behavior is promoted), and promotion (i.e., the tools used to promote the behavior).
6. Competition	It is imperative to understand what other things or behaviors are concurrently competing for the target recipients’ time and attention. Strategies can be developed to mitigate the impact of this competition.

^1^ Adapted from Andreasen [36], Fujijira et al., [37], Gordon et al., [13], and Luecking et al., [40].

**Table 4:** The Bristol Recommendations for Improving Physical Activity Communication.

Recommendations
1. Work with multi-disciplinary teams to develop physical activity communications and messages. Ensure that social marketing experts are included.
2. Develop a nuanced understanding of your target audience(s), and wherever possible work with your audience(s) to develop and tailor physical activity messages to their preferences.
3. Carefully consider message content, including use of language and whether to include physical activity guidelines; focusing on “moving more” may seem more feasible to the public. Jargon-free language is preferred.
4. Emphasize the benefits of physical activity and ensure that these include affective and social benefits, alongside physical and mental health benefits. Benefits will differ between audiences.
5. Identify influential and trustworthy individuals to deliver the physical activity message. It is often important that these individuals are relatable to the public–particularly for adult audiences (i.e., “look and talk like me”.
6. A multi-modal, visual approach is likely needed to widely disseminate the physical activity message. The preferred mechanism of delivery will differ between, and within, groups.
7. When creating physical activity messages, be mindful about the regular conflation of physical activity, sport and exercise. Ensure that communications do not increase public confusion about what constitutes physical activity.
8. Consider how resources could be pooled to create consistent physical activity messages to the public over a long period of time. Seek to reduce public confusion around physical activity recommendations.
9. Whilst official documentation (e.g., CMO physical activity guidelines/infographics) may not be created for public consumption, be wary that these documents–or excerpts of–may end up in the public domain and can reinforce public perceptions of physical activity (e.g., via MECC resources).
10. Critically reflect on current physical activity messages and campaigns in light of these findings and consider how they may be received, and interpreted, by under-served community groups.

## Supplementary Materials

The following are available online at https://www.mdpi.com/1660-4601/17/8/2782/s1. Supplement I: Overview of workshop and methods, Supplement II: Example observation template, Supplement III: “Let’s talk about physical activity” infographic.
