# Supplementary material for: “Let’s Talk about Physical Activity”: Understanding the Preferences of Under-Served Communities when Messaging Physical Activity Guidelines to the Public
Source: Int J Environ Res Public Health. 2020 Apr 17;17(8):2782. doi: 10.3390/ijerph17082782 (PMC7215851; doi:10.3390/ijerph17082782)
Supplement: Supplementary file 1 [file ijerph-17-02782-s001.zip › Supplement I - Overview of workshop and methods.docx]

**Overview of workshop and methods**

The outline was adapted to meet the needs of the community group being worked with. The activities and respective allocated times are only indicative. That said, each of the four workshops answered the questions set out in the activities below. In most cases, the four activities listed below required some form of physical activity in order to complete them.

| **Task** | **Time (Total)** |
| --- | --- |
| *Introductions*   - One project member to welcome all public contributors to the session - Project team members to introduce themselves to the group - Public contributors to introduce themselves to the group | 5 mins (5 mins) |
| *Overview of the session*   - Purpose of the workshop and what we are hoping to achieve - Overview of the workshop activities - Public contributors seen as an equal in this process | 5 mins (10 mins) |
| *Activity 1 – Understanding perceptions of physical activity*   - *Aim*: to understand what physical activity means to people, what it looks like to them, and whether it is important - *Method(s)*:   - Pass the ball: Participants stood in a circle and passed a ball between them. When the ball was passed to a new participant, they were encouraged by the facilitator to give examples of what physical activity looks like to them. Each participant had several opportunities to provide examples.   - Pin the post-it: Each participant was given a set of blank post-it notes. They were asked to write on these post-it notes what the benefits of physical activity were to them. Each person then went to the front of the room, and stuck their post-it notes on a big board. Participants started to group the benefits where similar ones were identified. Lastly, once the benefits had been grouped, the group were asked to rank the perceived importance of these benefits as a collective. Music was played throughout this part of the activity.   - Group discussion: The final part of this activity was to have an informal conversation with participants about the perceived importance of physical activity in their lives. | 15 mins (25 mins) |
| *Activity 2 – Knowledge of the CMO Physical Activity Guidelines*   - *Aim*: To briefly assess whether people are aware of the current physical activity guidelines for their age group - *Method(s)*:   - Ring a bell: The community artist brought a set of bells to the workshop, which were placed in front of participants in the centre of the room (for the purpose of this activity only). Participants were shown a series of prompt cards (one with the CMO guidelines, one with the Change4Life materials, and one with ThisGirlCan poster). If participants were familiar with the information on the prompt card, they were asked to ring a bell. Before showing the CMO Physical Activity Guideline prompt card, participants were asked if they knew that the government created recommendations for physical activity. If they did know that the government had recommendations, they were encouraged to state what they thought these were.   - Informal discussion followed each prompt card to understand more about where people had seen these materials, whether they liked them, whether they liked the tone etc…Participants were also asked whether there were any other health-related campaigns that they are familiar with. | 10 mins (35 mins) |
| *Activity 3 – Feedback on the 2019 update of CMO Physical Activity Guidelines*   - *Aim*: To present the CMO Physical Activity Guidelines which are relevant to their demographic and to gather their feedback on the feasibility of achieving this. - *Method(s)*:   - Small group work: Participants were broken up into small sub-groups. Each sub-group was given an excerpt of the age-specific CMO Physical Activity Guidelines (*e.g.,* adults were given the physical activity guidance for adults aged 18-65 years old). Groups were given time to discuss the following: 1) who is telling you this information? 2) where are you seeing this information?, 3) who is the information being targeted at?4) are the guidelines realistic? and, 5) which terms aren’t understandable? Groups were asked to feedback.   - Bin it: Participants were encouraged to note down, on post-it notes, the terminology within the guideline that they didn’t understand or didn’t like. They were then asked to scrunch up their post-it note and put it in a bin. As they put these terms in the bin, they were asked to state why they did not like these terms. | 20 mins (55 mins) |
| Break | 10 mins (65 mins) |
| *Activity 4 – How to communicate the CMO Physical Activity Guidelines to us*   - *Aim*: a) to create a series of messages that would help communicate the physical activity guideline(s) to their demographic, and b) to identify the most appropriate mechanisms to share these physical activity messages. - *Method(s)*:   - Design a physical activity message: The small groups were reconvened to collectively think about how to improve the communication of physical activity guidelines to the public. This activity was positioned as a challenge, whereby the groups work together to develop an effective physical activity message that would encourage people like them to undertake more physical activity. There were four key questions within this activity: 1) who should give the message? 2) what format should the message be given in? 3) what about the tone of the words? and 4) what should the message be? The groups were led through these four questions by the facilitator. At the end of the session, each group summarised their discussion, stuck this summary on a visual notice board, and then talked through their ideas with the rest of the group. | 50 mins (115 mins) |
| *Summary and close*   - Summarise the main points of the workshop - Restate how the information generated in the workshop will feed into the overarching project - Thank everyone for their participation | 5 mins (120 mins) |

**Images of the workshop content**


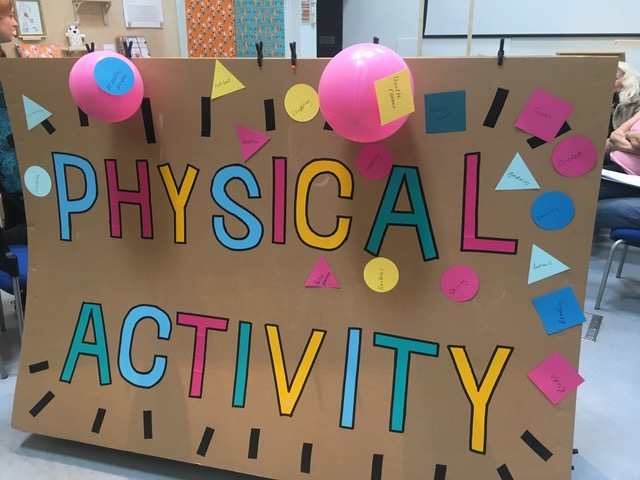


Image(s) 1: Notice board used in activity 1


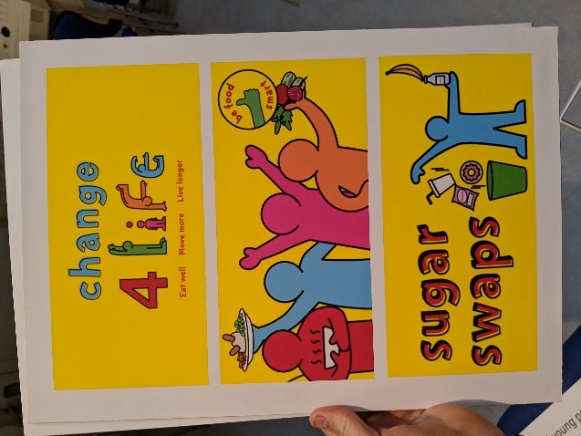

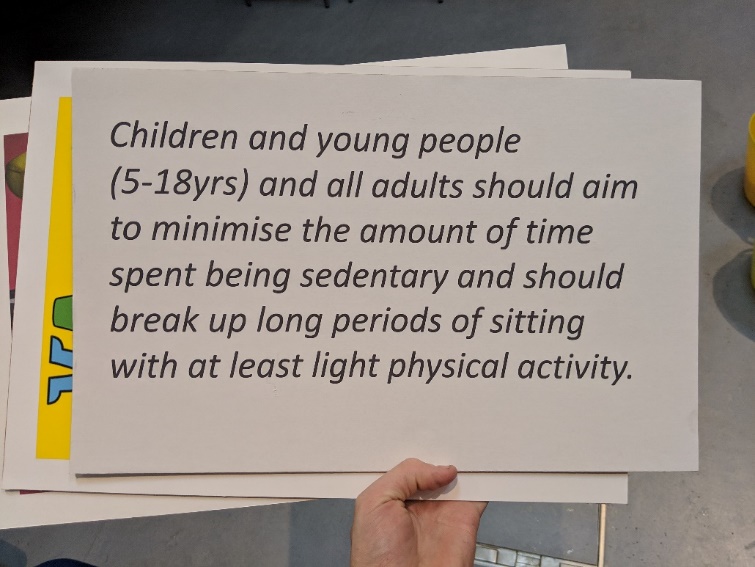

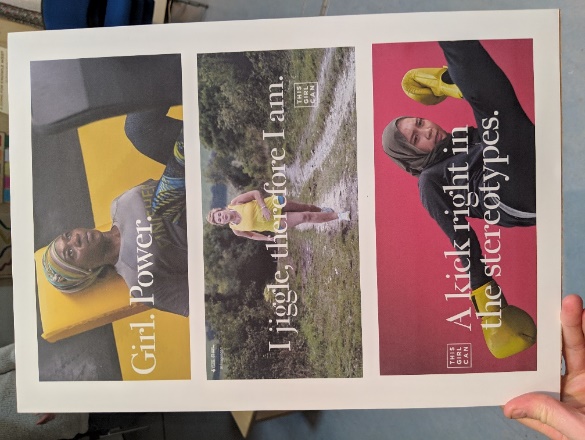


Image(s) 2-4: Prompt cards for "ring a bell"


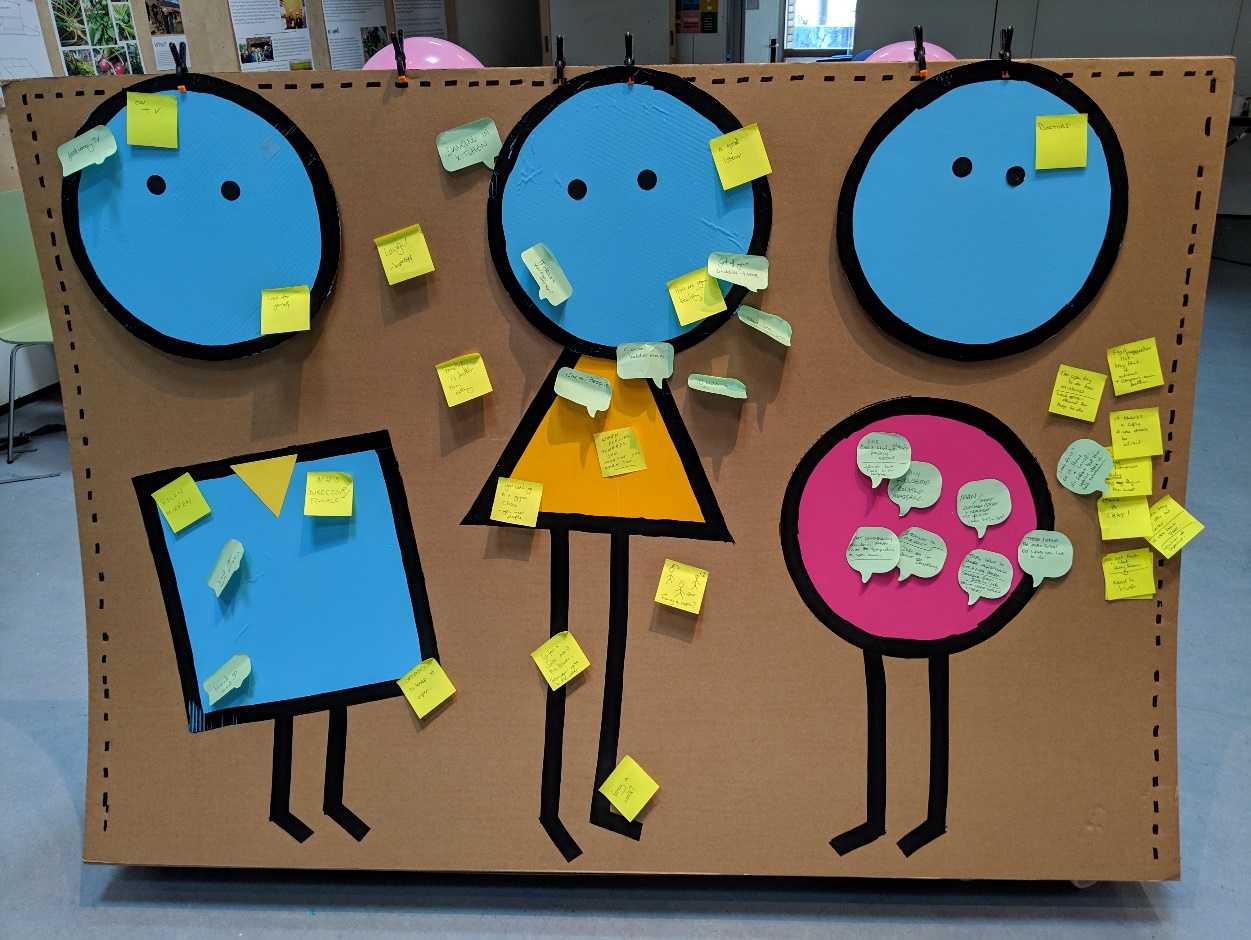


Image(s) 5: Notice board used in activity 4
