# Supplementary material for: “Let’s Talk about Physical Activity”: Understanding the Preferences of Under-Served Communities when Messaging Physical Activity Guidelines to the Public
Source: Int J Environ Res Public Health. 2020 Apr 17;17(8):2782. doi: 10.3390/ijerph17082782 (PMC7215851; doi:10.3390/ijerph17082782)
Supplement: Supplementary file 1 [file ijerph-17-02782-s001.zip › Supplement II - Example Observation Template.docx]

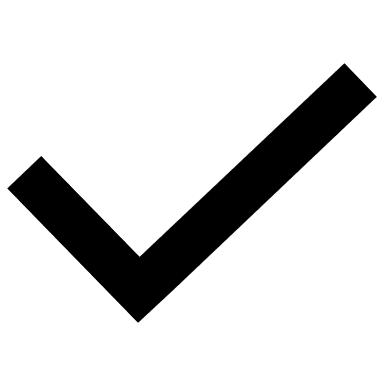
**Data Collection Checklist**

1. Gathered all completed consent forms (and assent forms for CYP group)
2. Completed the observation template (this document)*
   **Main document which we will base the majority of our analysis on.*
3. Used audio-recorders to capture group-based conversations**
   ***We can ask our participants to control the audio-recorders (if possible), and capture
   any small group-based conversation (one recorder per sub-group). It isn’t necessary to audio-
   record the whole workshop as sound quality will be impaired.*
4. Gathered anonymised demographic information on attendees***
   ****Please ask all participants to complete the demographics form at the end of the
   session. This is voluntary and participants do not need to provide their name. Only
    asked to provide: Age, Gender, Ethnicity, and first 4 digits of postcode. Enables us to describe
   who attended.*
5. Photos of outputs created throughout the workshop

6. Provide all participants with 2 X £10 gift vouchers as a thank you

**Observation Template**

**Observer**:

**Time / date / location**:

**Group:**

**Number of attendees**:

There are five sections to this observation template, and notes should relate to: 1) the four workshop activities; 2) the workshop as a whole; 3) the interaction between participants; 4) the workshop facilitation [if needed]; and 5) your reflections on the workshop. There is also space at the end of this document for any additional notes.

**Section 1: Workshop Activities**

Within the four sections below, we are predominantly interested in capturing notes on the discussion that participants are having and their responses to the questions asked by the facilitators. Please make notes on the content of the discussion, and key phrases that are being used, and any additional interactions that you hear between participants.

***Activity 1* – Perceptions of PA and its benefits**

1.1 What are the group’s perceptions around what PA is?

1.2 What do they say are the benefits associated with PA?

1.3 Is PA important to them and their group?

***Activity 2* – Current awareness and understanding of the CMO PA Guidelines**

1.4 How many participant know that there are recommendations for how much PA we should do?

1.5 How many participant state that they know what the guidelines are for their age group?

1.6 Any conversation around this?

1.7 If participant state that they know the guidelines, what do they think they are? (e.g. 5 X 30 minutes PA / week)

1.8 Any additional comments on participant’s awareness of PA guidelines?

***Activity 3* – Feedback on the current CMO PA Guidelines**

1.9 Regarding the PA guideline for their age group, what are the reflections and comments from the group on this?

1.10 Do participant understand the guideline(s)?

1.11 Is it realistic and achievable for them to meet the recommendation?

***Activity 4* – Communicating PA Guidelines to our demographic**:

1.12 What do participant think would be a more effective means of communicating the PA guidelines to their population group? In terms of (links to University of Edinburgh work – see appendix 1):

Note, the purpose of the message should be to help / motivate people become more active.

1.12.1 What should the *content of the message* be? (e.g. telling people about how much PA to do, why PA is important, or how to be more active). Note, content may be positively or negatively framed (gains and losses) and may be generalised or customised to specific groups.

1.12.2 How should the *message be communicated*? (i.e. what mechanisms and mediums should be used to share the message?). Consider both the *formatting* of the message (e.g. length of message or personalised content) and the *mode of deliver*y (e.g. how is message shared, targeted at groups, in which settings, how often is message shared). Also consider *who should be the messenger* (e.g. GPs, HCPs, athlete, community champion, peer etc…).

1.13 What is the feedback on alternative types of messaging campaigns (e.g. Change4Life, ThisGirlCan, VERB etc…)?

**Section 2: Workshop as a whole**

2.1 Any additional notes and observations on the delivery and interaction with the workshop as a whole?

2.3 How did the activities in the workshop link together? Did they?

**Section 3: Interaction between attendees**

3.1 Do the participants seem to know each other prior to workshop? Any additional comments on this?

3.2 Any dominant individuals influencing the workshop? If so, what impact on the session?

3.3 Are participant getting opportunity to talk / express themselves? Any comments?

3.4 If issues around the group dynamic arose, how was this managed?

**Section 4: Facilitation**

4.1 Are there any key points to note about the facilitation of the workshop, and whether this influenced how participant engaged with the activities or the responses they provided?

**Section 5: Researcher reflections**

5.1 What went well within the workshop and why? (i.e. strengths)

5.2 What didn’t work too well and why? Can this be improved prior to next workshop? (i.e. weaknesses)

5.3 Any other salient points of the workshop?

5.4 Did your presence have an impact on the workshop? If so, what impact or influence do you think you had?
