# Supplementary material for: “Let’s Talk about Physical Activity”: Understanding the Preferences of Under-Served Communities when Messaging Physical Activity Guidelines to the Public
Source: Int J Environ Res Public Health. 2020 Apr 17;17(8):2782. doi: 10.3390/ijerph17082782 (PMC7215851; doi:10.3390/ijerph17082782)
Supplement: Supplementary file 1 [file ijerph-17-02782-s001.zip › Supplement III - Infographic - Let's talk about physical activity.pdf]

10 ways to improve your physical activity communication: the Bristol recommendations

1. Work with multi-disciplinary teams, and ensure (social) marketers are involved.
2. Understand, and work with, your target audience to develop tailored messages.
3. Keep language simple. You may not need to include the physical activity guidelines!

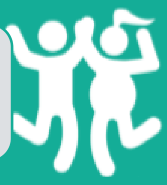

4. Promote the benefits of physical activity, including physical, mental and <u>social</u> health.
5. Identify influential <u>and</u> trustworthy individuals to deliver the message.

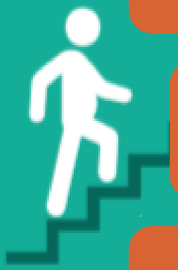

6. Use different mechanisms (e.g. posters, social media, TV) to spread a consistent message.
7. Terminology matters. Physical activity is more than just exercise and sport.
8. Consider how resources can be pooled between organisations to promote a consistent message.
9. Documents for health care professionals (e.g. infographics) are not designed for the public.

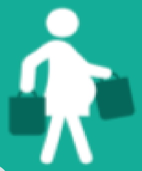

10. Reflect on current physical activity messages, and how they might be interpreted by the public.

Source: Nobles, J.; Thomas, C.; Banks Gross, Z. *et al.,* “Let’s talk about physical activity”: understanding the preferences of under-served communities when communicating physical activity messages to the public. *Int J Env Res Pub Health* **2020.**

KWMC KNOWLE WEST MEDIA CENTRE · NIHR | Applied Research Collaboration West
